# Histology-Correlated FTIR Chemical Imaging of Fungal Infection-Associated Tissue Compartments in Human Skin: A Proof-of-Concept Study

**DOI:** 10.3390/diagnostics16172789

**Published:** 2026-08-30

**Authors:** Maximilian Lammer, Paul Bellmann, Matthias Schmuth, Verena Moosbrugger-Martinz, Bernhard Zelger, Bettina Zelger, Birgit Moser, Petra Hatzer-Grubwieser, Claudia Wöss, Roland Stalder, Lisa-Maria Zenz, Michaela Lackner, Christian Wolfgang Huck, Miranda Klosterhuber, Johannes Dominikus Pallua

**Affiliations:** 1 Department of Dermatology, Venereology and Allergy, Medical University Innsbruck, 6020 Innsbruck, Austria; maximilian.lammer@i-med.ac.at (M.L.); paul.bellmann@i-med.ac.at (P.B.); matthias.schmuth@i-med.ac.at (M.S.); verena.martinz@i-med.ac.at (V.M.-M.); birgit.moser@tirol-kliniken.at (B.M.); 2 Private Dermatological Practice, Specialized in Dermatopathology, Mariahilfpark 1/6, 6020 Innsbruck, Austria; bernhard.zelger@gmail.com; 3 Institute of Pathology, Neuropathology, and Molecular Pathology, Medical University of Innsbruck, Muellerstrasse 44, 6020 Innsbruck, Austria; bettina.zelger@i-med.ac.at; 4 Institute of Legal Medicine, Medical University of Innsbruck, Muellerstraße 44, 6020 Innsbruck, Austria; petra.hatzer@i-med.ac.at (P.H.-G.); claudia.woess@i-med.ac.at (C.W.); 5 University of Innsbruck, Institute of Mineralogy and Petrography, 6020 Innsbruck, Austria; roland.stalder@uibk.ac.at; 6 Institute for Hygiene and Medical Microbiology, Medical University of Innsbruck, 6020 Innsbruck, Austria; lisa-maria.zenz@i-med.ac.at (L.-M.Z.); michaela.lackner@i-med.ac.at (M.L.); 7 Institute of Analytical Chemistry and Radiochemistry, University of Innsbruck, 6020 Innsbruck, Austria; christian.w.huck@uibk.ac.at; 8 Department of Orthopaedics and Traumatology, Medical University Innsbruck, 6020 Innsbruck, Austria; miranda.klosterhuber@i-med.ac.at

**Keywords:** FTIR chemical imaging, fungal skin infection, dermatopathology, infection-associated spectral signatures, Grocott methenamine silver staining, periodic acid–Schiff staining, keratosis, vital epidermis, CytoSpec clustering, infrared spectroscopic imaging

## Abstract

**Background/Objectives:** Fungal skin infections are commonly assessed using clinical examination and conventional histopathology, including hematoxylin-eosin (HE), periodic acid–Schiff (PAS), and Grocott methenamine silver (GMS) staining. However, these methods provide limited spatially resolved biochemical information. This proof-of-concept study investigated whether Fourier transform infrared (FTIR) chemical imaging can identify biochemical patterns associated with histologically defined fungal infection-associated tissue compartments in human skin. **Methods:** Archived formalin-fixed, paraffin-embedded skin samples with histological evidence of fungal infection were investigated. The study group comprised 19 patients, of whom 12 fulfilled the histological and technical eligibility criteria for quantitative FTIR analysis. These 12 independent biological cases yielded 46 histologically defined regions of interest (ROIs), comprising 16 fungal infection-associated ROIs, 14 keratosis/keratinised tissue ROIs, and 16 vital epidermis ROIs. ROI assignment was guided by corresponding HE-, PAS-, and GMS-stained sections. **Results:** Fungal infection-associated tissue compartments showed partially distinct spectral characteristics compared with vital epidermis and keratinised tissue. The most prominent exploratory differences occurred within the 900–1300 cm^−1^ fingerprint region. Case-level statistical analysis showed significant differences between fungal infection-associated tissue and vital epidermis at approximately 1185 and 1240 cm^−1^ after false discovery rate correction, whereas substantial overlap with keratinised tissue remained. Case-level PCA retained tissue-associated spectral structure after biological aggregation, although fungal infection-associated and keratinised tissue showed partial overlap. Unsupervised clustering further demonstrated spatially coherent spectral compartments corresponding to histologically identifiable tissue structures. **Conclusions:** FTIR chemical imaging may complement conventional histopathology by providing label-free, spatially resolved biochemical information on fungal infection-associated tissue compartments. Because fungal elements are embedded within surrounding keratinised and epithelial tissue, the observed spectral characteristics should be interpreted as exploratory infection-associated tissue signatures rather than fungal-specific diagnostic biomarkers. Larger independent studies with case-wise validation are required before diagnostic application can be considered.

## 1. Introduction

Cutaneous fungal infections are among the most frequent infectious diseases encountered in dermatology and dermatopathology. They may be caused by dermatophytes, yeasts, and non-dermatophyte moulds and commonly involve keratin-rich structures such as the stratum corneum, hair follicles, and nails [1,2]. Although many superficial fungal infections are not life-threatening, they are clinically relevant because they can mimic inflammatory dermatoses, persist despite therapy, recur, or become difficult to treat when diagnosis is delayed or when resistant organisms are involved [1]. In routine practice, the diagnostic workflow usually combines clinical assessment with direct microscopy, culture, histopathology, and increasingly, molecular methods. However, each of these approaches has limitations. Potassium hydroxide microscopy and fungal culture are complementary rather than interchangeable tests, with microscopy often providing rapid but operator-dependent information and culture enabling organism identification but requiring viable organisms and longer turnaround times [3].

Histopathology remains particularly important when fungal infection is clinically unsuspected, when lesions are atypical, or when tissue invasion and the inflammatory tissue response need to be assessed. Hematoxylin and eosin (HE) staining provides morphological context, whereas periodic acid–Schiff (PAS) and Grocott methenamine silver (GMS) stains are widely used to highlight fungal cell wall structures [2]. Nevertheless, histological diagnosis is not always straightforward. Fungal elements can be sparse, masked by keratin, or difficult to distinguish from artefacts. Moreover, several studies have shown that histological “clues” of tinea, such as parakeratosis, neutrophils in the stratum corneum, or compact orthokeratosis, are not sufficiently reliable on their own and require confirmatory fungal stains [4]. Studies comparing HE, PAS, and GMS have further suggested that special stains improve the detection of fungal elements, although their relative performance can vary depending on specimen type, fungal burden, and tissue context [5,6]. Thus, while conventional histology is indispensable, it primarily provides morphology-based information and does not directly characterise the spatially resolved biochemical composition of infected tissue. Reported diagnostic performance of conventional methods varies substantially according to specimen type, disease setting, and the reference standard used. In a pooled analysis of tinea pedis, potassium hydroxide (KOH) smear showed a sensitivity of 73.3% and specificity of 42.5%, whereas fungal culture showed a sensitivity of 41.7% and specificity of 77.7% when clinical assessment was used as the reference standard [3]. In the histopathological assessment of invasive fungal wound infections, PAS and GMS staining showed sensitivities of 56% and 85%, respectively, for fungal detection when the presence of fungal elements identified by any histopathological method was used as the reference [6]. These findings illustrate that diagnostic-performance estimates are strongly context-dependent and should not be transferred directly between superficial fungal infection, invasive fungal disease, and histological tissue analysis.

Fourier transform infrared (FTIR) spectroscopy and FTIR chemical imaging offer complementary approaches for measuring the intrinsic molecular vibrations of biological samples. In biological tissues, infrared spectra contain information from major biochemical classes, including proteins, lipids, nucleic acids, carbohydrates, and extracellular matrix components [7]. When combined with microscopy, FTIR spectroscopic imaging enables label-free, spatially resolved chemical mapping of tissue sections and has been increasingly explored for digital histopathology, tissue classification, and disease recognition [8,9,10]. A major advantage of this approach is that spectral information can be evaluated using both targeted single-band or spectral-region analysis and multivariate methods. Selected wavenumbers or spectral regions can be visualised as chemical maps, whereas chemometric approaches such as principal component analysis and unsupervised clustering analyse patterns across multiple spectral variables simultaneously. This allows complex tissue compartments to be visualised not only by morphology but also by biochemical composition.

In the field of infectious diseases and mycology, infrared spectroscopy has shown promise for identifying and differentiating fungal organisms. FTIR-based approaches have been reported for the rapid identification of Candida and dermatophyte species and for the diagnosis of onychomycosis, supporting the concept that fungal organisms and infected keratinised tissues contain diagnostically useful infrared spectral signatures [11,12]. However, most previous studies have focused on microbial isolates, nail material, or bulk measurements rather than histologically correlated chemical imaging of fungal structures directly within skin tissue sections. This distinction is important because fungi in skin are often embedded within or adjacent to keratinised tissue, creating a major analytical challenge: spectral patterns associated with fungal infection must be distinguished from the strong biochemical contribution of keratin, lipids, and surrounding epidermal structures.

Importantly, quantitative diagnostic-classification performance has also been reported for label-free infrared spectroscopy in other fungal disease settings. In an ATR-FTIR study of onychomycosis, prediction models achieved a correct classification of 96.9% for uninfected nails and nails infected with dermatophytes, non-dermatophytes, or yeasts. Correct classification rates of 91.0%, 97.7%, and 98.6% were additionally reported for discrimination among dermatophyte, non-dermatophyte, and yeast genera or species, respectively [11]. However, these results were obtained using culture-based, ex vivo nail, and in vivo nail models and should not be directly extrapolated to histologically heterogeneous FFPE skin sections.

This issue represents a central methodological question in FTIR-based dermatopathology. On the one hand, fungal cell walls contain carbohydrate-rich components that are expected to contribute to the infrared fingerprint region. On the other hand, keratinised tissue also emits strong infrared signals, particularly in regions associated with protein, lipid, and carbohydrate-like vibrations. Therefore, spectral differences observed in infected skin cannot automatically be interpreted as fungal-specific. A robust analysis must include keratosis or keratinised tissue as an independent comparator and must validate spectral findings against histological reference stains. Recent work on FTIR microscopy in parasitic skin disease has demonstrated that infrared imaging can detect biologically meaningful tissue and parasite-associated structures in histological sections, supporting the feasibility of this approach for dermatopathological applications [13].

The present study investigated whether FTIR chemical imaging can identify spatially coherent biochemical patterns associated with fungal infection in human skin tissue sections. Histologically characterised samples were analysed using HE, PAS, and GMS staining, in combination with FTIR spectroscopic imaging. Regions of interest were assigned to histologically defined fungal infection-associated tissue compartments, keratinised tissue, and vital epidermis, and spectral differences were evaluated using chemical maps, mean spectra, principal component analysis, and CytoSpec-based clustering. We hypothesised that fungal infection-associated regions would show spectral characteristics that differ from those of vital epidermis and keratosis, particularly in the infrared fingerprint region. The aim was to assess the feasibility of FTIR chemical imaging as a complementary, label-free method for the spatially resolved biochemical characterisation of histologically defined fungal infection-associated tissue compartments, while interpreting any observed spectral differences as exploratory infection-associated patterns rather than validated diagnostic biomarkers.

## 2. Materials and Methods

### 2.1. Study Design and Tissue Specimens

This proof-of-concept study was designed to evaluate whether Fourier transform infrared (FTIR) chemical imaging can identify spatially resolved biochemical patterns associated with fungal infection in human skin tissue sections. Nineteen formalin-fixed, paraffin-embedded (FFPE) human skin tissue cases with histological evidence of fungal infection constituted the initial retrospective study group. Cases were included when sufficient tissue was available for serial histological staining and FTIR spectroscopic imaging, and when fungal infection-associated regions could be identified by conventional histology.

The initial study group comprised 19 patients with histological evidence of fungal skin infection. Of these, 12 cases fulfilled all requirements for quantitative FTIR analysis, namely sufficient residual FFPE tissue for preparation of the required serial histological and FTIR sections and the presence of a fungal infection-associated tissue compartment that could be reliably identified by conventional histology and anatomically correlated with the unstained FTIR section. The remaining seven cases were not included in the quantitative FTIR dataset because they did not fulfil all of these histological and/or technical requirements. Consequently, the final quantitative FTIR dataset comprised 12 independent biological cases.

The analysis focused on three histologically defined tissue compartments: fungal infection-associated regions, keratinised tissue/keratosis, and vital epidermis.

The study was performed in accordance with institutional and ethical requirements for the use of archived human tissue.

### 2.2. Histological Staining

Serial sections were prepared from selected FFPE tissue blocks according to routine dermatopathology procedures. Sections used for histological evaluation were mounted on glass slides and stained with hematoxylin and eosin (HE) to assess general tissue architecture, inflammatory changes, epidermal morphology, and keratinised compartments. Special stains were used to support the detection and localisation of fungal structures. Periodic acid–Schiff (PAS) staining and Grocott methenamine silver (GMS) staining were applied to visualise fungal cell wall-associated structures and to guide the assignment of fungal infection-associated regions of interest.

Histological evaluation was performed by correlating HE morphology with the PAS and GMS findings. Fungal infection-associated regions were defined as histological tissue compartments containing or anatomically corresponding to PAS- and/or GMS-positive fungal structures. The term “fungal infection-associated” was deliberately used because these compartments may comprise fungal elements together with keratinised tissue, epithelial components, and the surrounding local tissue microenvironment and therefore do not represent isolated fungal material. Keratosis was defined as keratinised or hyperkeratotic tissue without a dominant histologically confirmed fungal focus. Vital epidermis was defined as morphologically preserved viable epidermal tissue. These histological categories served as the biological reference for FTIR region-of-interest selection and interpretation.

Histological assessment and definition of the reference tissue compartments were performed by two experienced pathologists (Bettina Zelger and Bernhard Zelger) together with an experienced dermatologist (Matthias Schmuth). Their combined assessment served as the histopathological reference for subsequent FTIR region-of-interest selection and interpretation.

### 2.3. Digital Slide Imaging and Histological Correlation

Stained histological sections were digitised using a Pannoramic SCAN digital slide scanner (3DHISTECH, Budapest, Hungary) equipped with a plan-apochromatic objective at 20× magnification. Whole-slide images were examined and annotated using Pannoramic Viewer software (version 1.15.4; 3DHISTECH, Budapest, Hungary). Digital images were used to identify tissue compartments, localise fungal structures, and guide the correlation between histology and FTIR chemical maps.

Because FTIR imaging was performed on unstained sections and histology was performed on adjacent or serial stained sections, exact pixel-level registration was not assumed. Instead, histological and FTIR data were compared by anatomical and morphological landmarks, including epidermal contours, keratinised layers, follicular or infundibular structures, and fungal foci identified by PAS or GMS staining.

### 2.4. Preparation of Sections for FTIR Spectroscopic Imaging

For FTIR spectroscopic imaging, unstained 5 µm thick tissue sections were prepared from the same FFPE blocks and mounted on calcium fluoride (CaF_2_) slides suitable for transmission-mode infrared microscopy. Sections were deparaffinised prior to FTIR measurement using xylol for 45 min, followed by 100% ethanol for 5 min, then 70% ethanol for 5 min, and finally 50% ethanol for 5 min.

These were then dried in an incubator at 60 °C for 15 min, followed by equilibration at room temperature (20–22 °C) for 30 min before FTIR acquisition. This drying procedure was used to facilitate the removal of residual solvent and moisture after deparaffinisation and to obtain stable dry tissue sections for transmission-mode FTIR imaging. The protocol was applied identically to all samples and has previously been used for the FTIR imaging of deparaffinised human skin tissue sections [13]. The subsequent room-temperature equilibration step was included to ensure that spectra were acquired only after the sections had returned to ambient measurement conditions. Although the heating step was brief and applied uniformly to all samples, subtle temperature-dependent effects on hydration-sensitive or protein-associated spectral features cannot be completely excluded and were therefore considered a potential pre-analytical limitation.

### 2.5. DNA Extraction, Quantification, PCR, and Sequencing Attempt

To obtain complementary molecular information on the causative fungal species, DNA extraction from FFPE tissue sections was attempted in collaboration with the molecular microbiology laboratory. For each case, two 10-µm FFPE sections were processed jointly. Following deparaffinisation, DNA was extracted using the QIAamp DNA Investigator Kit on the automated EZ2 platform (Qiagen, Hilden, Germany) according to the laboratory protocol, with a final elution volume of 30 µL. Extracted DNA was quantified and assessed for purity by spectrophotometric absorbance measurements. Fungal DNA amplification targeted the internal transcribed spacer 2 (ITS2) region using the ITS3 primer (5′-TGT AAA ACG ACG GCC AGT GCA TCG ATG AAG AAC GCA GC-3′) and the ITS4 primer (5′-CAG GAA ACA GCT ATG ACC TCC TCC GCT TAT TGA TAT GC-3′). PCR reactions were performed in a total volume of 25 µL containing 12.5 µL Taq 2 × Master Mix (New England Biolabs, Ipswich, MA, USA; M0270L), 1 µL ITS3 primer (10 µM), 1 µL ITS4 primer (10 µM), 2 µL template DNA, and 8.5 µL nuclease-free water. Cycling conditions consisted of an initial denaturation at 95 °C for 2 min, followed by 40 cycles of denaturation at 95 °C for 30 s, annealing at 52 °C for 30 s, and extension at 72 °C for 1 min, followed by a final extension at 75 °C for 5 min. PCR products were evaluated by gel electrophoresis. Available PCR products were purified using the Exo-CIP Rapid PCR Cleanup Kit (New England Biolabs; E1050L) and submitted for sequencing using the Mix2Seq service (Eurofins Genomics Germany GmbH, Ebersberg, Germany). Species-level identification was intended by comparison of the resulting sequence data using NCBI BLAST (National Center for Biotechnology Information, Bethesda, MD, USA) and MycoBank (Westerdijk Fungal Biodiversity Institute, Utrecht, The Netherlands). Only limited amplification was obtained, with few visible PCR products on gel electrophoresis. Sequencing of the available PCR products was nevertheless attempted; however, no sequence data of sufficient quality for reliable species-level identification were obtained. Following renewed purification of the DNA samples, DNA concentration and purity measurements were less favourable, and additional PCR attempts did not yield suitable amplification products. Consequently, molecular species identification remained inconclusive, and database-based species assignment using NCBI BLAST or MycoBank was not performed. Molecular findings were therefore not used for case classification or ROI definition. Histological identification based on HE, PAS, and/or GMS staining remained the biological reference for defining fungal infection-associated tissue compartments.

### 2.6. FTIR Spectroscopic Imaging

FTIR spectroscopic imaging was performed in transmission mode using a Bruker Vertex 70 FTIR spectrometer (Bruker Optik GmbH, Ettlingen, Germany) coupled to a Hyperion 3000 infrared microscope. The microscope was equipped with a 64 × 64 mercury cadmium telluride (MCT) focal plane array (FPA) detector and a 15× infrared objective (NA = 0.4). Spectra were acquired over the spectral range from 3800 to 900 cm^−1^ at a spectral resolution of 8 cm^−1^ using 16 co-added sample scans. Background spectra were acquired using 16 co-added scans before each measurement and were used by the Bruker system for instrumental background correction. No pixel binning was applied. The acquisition configuration provided a projected spatial sampling of approximately 2.7 × 2.7 µm^2^ per pixel and a field of view of approximately 173 × 173 µm^2^. Measurements were performed at room temperature under dry-air purge conditions. Data acquisition and initial spectral handling were performed using OPUS software version 7.2 (Bruker Optik GmbH).

### 2.7. Spectral Preprocessing and FTIR Chemical Mapping

FTIR spectral data were processed using a standardised preprocessing workflow. Instrumental background correction was performed during FTIR acquisition by the Bruker Hyperion/Vertex system using a background spectrum acquired before each measurement. No additional measured background or reference spectrum was subtracted during subsequent spectral preprocessing.

Preprocessing was applied in the following order. First, the spectral interval from 1796.5 to 2789.1 cm^−1^ was removed, thereby retaining the fingerprint and C–H stretching regions. Baseline correction was then performed using a rubber-band algorithm with positive peak direction. Within this procedure, the calculated rubber-band baseline was subtracted from each spectrum using the “Background Action: Subtract” setting. This operation represents mathematical baseline correction and is distinct from the instrumental background-spectrum correction performed during FTIR acquisition. Following baseline correction, spectra were vector-normalised. No Gaussian smoothing or area normalisation was applied.

The same preprocessing sequence and parameter settings were applied consistently to spectra used for downstream spectral analyses. Quasar software version 1.7.0 was used for spectral preprocessing and chemical-map generation, and Orange Data Mining version 3.40.0 with the Orange Spectroscopy add-on was used for complementary multivariate data analysis.

Univariate chemical maps were generated to visualise the spatial distributions of selected spectral bands and spectral regions. For single-wavenumber maps, the spectral variable closest to the predefined target wavenumber was selected using the “Closest value” option. The main spectral regions evaluated included the fingerprint region between 900 and 1300 cm^−1^, with emphasis on 900–1120 cm^−1^, approximately 1085 cm^−1^, 1120–1200 cm^−1^, approximately 1185 cm^−1^, 1200–1300 cm^−1^, and approximately 1240 cm^−1^. Additional maps were generated from the Amide II region around 1500–1580 cm^−1^, the Amide I region around 1620–1680 cm^−1^, and the C–H stretching region between 2800 and 3000 cm^−1^, including approximately 2922 cm^−1^.

### 2.8. Region-of-Interest Selection and Spectral Extraction

Regions of interest were selected by anatomical and morphological correlation of the unstained FTIR section with the corresponding HE-, PAS-, and GMS-stained serial sections. Three ROI classes were defined: fungal infection-associated tissue compartments, keratosis/keratinised tissue, and vital epidermis. Fungal infection-associated ROIs were defined at the level of the histologically affected tissue compartment rather than as regions containing spectroscopically pure fungal material. These were selected from areas anatomically corresponding to PAS- and/or GMS-positive fungal structures identified in the serial histological sections. Because FTIR imaging was performed on an unstained serial section and exact pixel-to-pixel registration between the FTIR section and stained histological sections was not possible, the selected ROIs could also include immediately contiguous keratinised or epithelial tissue belonging to the same histologically affected compartment. Consequently, spectra assigned to the fungal infection-associated class may contain variable contributions from fungal structures, keratinised tissue, surrounding epithelial components, and the local tissue microenvironment. These spectra were therefore interpreted throughout the study as fungal infection-associated tissue spectra and not as spectra derived exclusively from fungal organisms. Keratosis ROIs were selected from keratinised or hyperkeratotic tissue without a dominant PAS- or GMS-positive fungal focus in the corresponding histological region. Vital epidermis ROIs were selected from morphologically preserved viable epidermal compartments without a dominant histologically identifiable fungal focus. Because individual fungal structures may approach or fall below the wavelength-dependent effective spatial resolution of FTIR imaging, individual spectra were not assumed to represent isolated fungal structures but were interpreted at the level of the histologically defined fungal infection-associated tissue compartment. The final dataset comprised 46 ROI datasets from 12 independent biological cases and included 16,498 individual FTIR spectra. Sixteen ROI datasets were assigned to fungal infection-associated tissue compartments (6841 spectra; 12 cases), 14 to keratosis/keratinised tissue (1122 spectra; 10 cases), and 16 to vital epidermis (8535 spectra; 12 cases). Some cases contributed more than one ROI of the same tissue class. Individual spectra and ROI datasets were therefore considered hierarchically nested within biological cases and were not treated as independent biological replicates. No additional automated spectrum-quality threshold based on signal-to-noise ratio, absorbance intensity, or spectral quality indices was applied after ROI selection. Spectra included in the analysis were those contained within the histologically defined ROIs and successfully acquired by the FTIR imaging system; preprocessing was subsequently applied uniformly to all included spectra.

### 2.9. Mean Spectra and Exploratory Spectral Analysis

Class-wise spectral summaries were calculated using a hierarchical averaging procedure. Individual preprocessed spectra were first averaged within each ROI to obtain one ROI-level mean spectrum. Where more than one ROI of the same tissue class was available from the same biological case, the corresponding ROI-level mean spectra were further averaged with equal ROI weighting to generate one case-level mean spectrum per tissue class. This resulted in 12 fungal infection-associated, 10 keratosis/keratinised tissue, and 12 vital epidermis case-level spectra. Class-wise mean spectra and standard deviations were subsequently calculated across these case-level spectra. Spectral differences were assessed descriptively across the analysed spectral range, with particular emphasis on the 900–1300 cm^−1^ fingerprint region, which contains overlapping contributions from carbohydrates, proteins, nucleic acids, phosphate-containing biomolecules, and other tissue components. Difference spectra were used exploratively to highlight spectral regions contributing to tissue class-associated variation. These analyses were interpreted as exploratory tissue-compartment comparisons rather than as diagnostic classification.

### 2.10. Principal Component Analysis

Principal component analysis (PCA) was performed as an exploratory unsupervised method to visualise spectral relationships among the three histologically defined tissue classes. Spectra were processed using the same preprocessing workflow described above. PCA was evaluated at different levels of the hierarchical dataset to account for the nesting of individual spectra within ROIs and biological cases. For the ROI-level analysis, all preprocessed spectra belonging to an individual ROI were averaged to generate one mean spectrum per ROI. The resulting dataset comprised 46 ROI-level spectra, including 16 fungal infection-associated, 14 keratosis/keratinised tissue, and 16 vital epidermis spectra. For the case-level analysis, ROI-level mean spectra belonging to the same tissue class and biological case were further averaged where applicable, so that each case contributed a maximum of one mean spectrum per tissue class. The resulting case-level dataset comprised 12 fungal infection-associated, 10 keratosis/keratinised tissue, and 12 vital epidermis observations. This approach prevented cases containing larger numbers of individual FTIR spectra from receiving greater statistical weight in the PCA. PCA was performed separately for the 900–1800 cm^−1^ fingerprint region, the 2800–3000 cm^−1^ C–H stretching region, and the combined 900–1800 cm^−1^ and 2800–3000 cm^−1^ regions. PCA was performed without using tissue-class labels for model construction. Before PCA, variables were mean-centred without unit-variance autoscaling. The percentages of variance explained by the principal components were calculated, and the corresponding PC1 and PC2 loading spectra were examined to identify the spectral variables contributing most strongly to the observed data structure. The original individual-spectrum PCA was retained as a complementary visualisation of within-class spectral heterogeneity but was not interpreted as representing independent biological observations. The case-level PCA was considered the primary analysis for assessing whether the spectral structure persisted when individual biological cases contributed comparable weight. No cross-validation was applied because PCA was used exclusively as an exploratory unsupervised dimensional-reduction method and not as a predictive classification model.

### 2.11. Multivariate Image Analysis

Multivariate image analysis was performed using CytoSpec version 2.10.01 (64-bit). Unsupervised clustering was applied to selected FTIR datasets to investigate whether histologically defined tissue compartments formed spatially coherent spectral clusters. Three unsupervised clustering methods were used: k-means clustering (KMC), fuzzy c-means clustering (FCM), and hierarchical cluster analysis (HCA). These clustering approaches have previously been applied to FTIR spectroscopic imaging for unsupervised tissue segmentation and for the identification of spatially coherent biochemical compartments in histological sections [14,15,16].

KMC partitions spectra into a predefined number of mutually exclusive clusters by assigning each spectrum to the cluster with the nearest centroid. In contrast, FCM assigns graded membership values to multiple clusters, thereby allowing gradual spectral transitions between adjacent or biochemically heterogeneous tissue compartments to be represented. HCA organises spectra according to their pairwise similarity and represents their relationships in a hierarchical structure.

For representative cases, clustering solutions containing 6, 7, and 8 clusters were generated to explore the effect of cluster number on the spatial segmentation of histologically recognisable tissue compartments. The range of 6–8 clusters was selected empirically to provide sufficient spectral subdivision to represent the heterogeneous tissue architecture without excessive fragmentation of morphologically coherent regions. Cluster maps were compared visually with the corresponding HE-, PAS-, and GMS-stained serial sections.

Selection of the representative cluster solution was therefore based primarily on visual interpretability and spatial correspondence with histologically identifiable tissue compartments rather than on an objective cluster-validity index. Seven-class KMC and FCM solutions were selected for representative presentation because they provided the most interpretable balance between the separation of major tissue compartments and preservation of spatially coherent structures. Six-class solutions tended to merge some visually distinct tissue compartments, whereas eight-class solutions resulted in greater subdivision without providing an obvious improvement in histological interpretability. The clustering analysis was exploratory and was not intended to define an optimal or diagnostically validated number of tissue classes.

### 2.12. Data Analysis and Statistical Considerations

The study was designed as an exploratory proof-of-concept analysis and did not aim to establish diagnostic sensitivity, specificity, or clinical classification performance. Individual FTIR spectra were hierarchically nested within ROIs and biological cases and were therefore not considered independent biological observations. To account for this structure, descriptive spectral summaries and the primary PCA were performed after aggregation at the ROI and case levels. Each case contributed a maximum of one mean spectrum per tissue class to the case-level PCA.

Quantitative band-level analysis was additionally performed for the spectral features highlighted in the descriptive spectral analysis. The nominal bands at 1030, 1085, 1185, 1240, 1450, 1540, 1650, 2850, and 2922 cm^−1^ were represented by the closest available measured spectral variables. Statistical analysis was performed at the biological case level to account for the hierarchical structure of the dataset. Individual preprocessed spectra were first averaged within each ROI. Where multiple ROIs of the same tissue class were available from one biological case, the corresponding ROI-level means were further averaged with equal ROI weighting, so that each case contributed a maximum of one value per tissue class and spectral band.

Overall differences among fungal infection-associated tissue, keratosis/keratinised tissue, and vital epidermis were assessed using Friedman tests in the 10 cases containing all three tissue compartments. Pairwise within-case comparisons were subsequently performed using two-sided Wilcoxon signed-rank tests. Twelve paired cases were available for the comparison between fungal infection-associated tissue and vital epidermis, whereas 10 paired cases were available for comparisons involving keratosis. To account for multiple band-wise comparisons, Benjamini–Hochberg false discovery rate correction was applied across the nine evaluated spectral bands separately within each pairwise comparison and for the overall tests. Adjusted q-values < 0.05 were considered statistically significant. Because the investigated bands were selected based on the exploratory spectral analysis, these statistical comparisons were considered exploratory rather than confirmatory.

No supervised diagnostic classifier was developed in the present study. Consequently, cross-validation and predictive performance metrics were not applied to the exploratory PCA. Future diagnostic classification studies should use larger independent cohorts and strictly case-wise validation strategies, such as leave-one-case-out cross-validation, to ensure that spectra originating from the same patient cannot contribute to both model training and testing. No a priori power calculation was performed because this retrospective study was designed as an exploratory proof-of-concept investigation and did not test a predefined diagnostic-performance hypothesis. The sample size was determined by the availability of archived cases fulfilling the predefined histological and technical eligibility criteria. Accordingly, the study should be considered feasibility-driven rather than statistically powered for diagnostic validation.

## 3. Results

Our initial study group comprised 19 patients (12 men and 7 women) with a mean age of 67.89 years (range 18–90 years), all of whom had histological evidence of fungal skin infection. Of these 19 cases, 12 fulfilled the histological and technical eligibility criteria for quantitative FTIR analysis described in Section 2.1, whereas seven did not meet all requirements for preparation and histology-guided correlation of the FTIR dataset. Quantitative FTIR spectral analysis was therefore performed on 46 histologically defined ROI datasets derived from these 12 independent biological cases. The dataset comprised 16 fungal infection-associated ROIs, 14 keratosis/keratinised tissue ROIs, and 16 vital epidermis ROIs. Because some cases contributed more than one ROI of the same tissue class, ROI counts were higher than the corresponding numbers of independent cases.

### 3.1. Overview of the Analysed FTIR Imaging Dataset

A total of 46 region-of-interest (ROI) spectral datasets derived from 12 independent biological cases were included in the quantitative FTIR analysis, comprising 16,498 individual spectra. Sixteen ROI datasets were assigned to fungal infection-associated tissue compartments (6841 spectra; 12 cases), 14 to keratosis/keratinised tissue (1122 spectra; 10 cases), and 16 to vital epidermis (8535 spectra; 12 cases) (Table 1). Ten cases contributed all three tissue classes, whereas two cases contributed fungal infection-associated and vital epidermal ROIs but no separate keratosis ROI. Several cases contributed more than one ROI of the same tissue class; these ROIs were retained as separate observations for ROI-level analyses but were averaged within case and tissue class for case-level analyses.

### 3.2. Histology-Guided Identification of Fungal Infection-Associated Tissue Compartments

**Figure 1:**
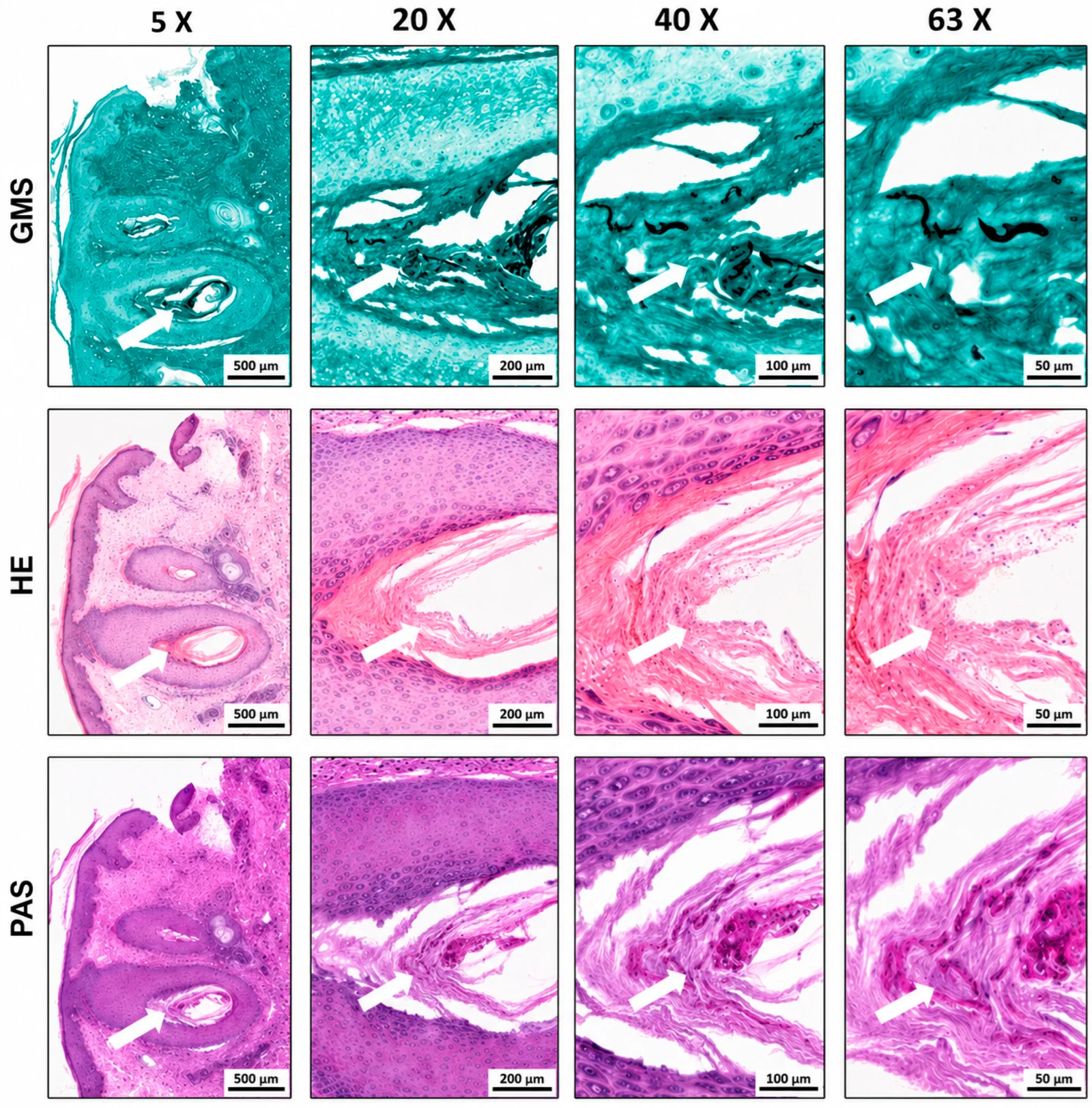
Histological reference staining of fungal infection-associated skin tissue. Representative serial sections of a fungal infection-associated skin lesion stained with Grocott methenamine silver (GMS), hematoxylin and eosin (HE), and periodic acid–Schiff (PAS). Images are shown at increasing magnification from left to right (5×, 20×, 40×, and 63×). GMS staining highlights black-stained fungal elements within keratinised tissue compartments, whereas HE demonstrates the general epidermal, keratinised, and stromal tissue architecture. PAS staining provides complementary visualisation of fungal and keratin-associated structures. White arrows indicate the corresponding fungal infection-associated region across staining modalities and magnifications. These histological reference images were used to guide region-of-interest selection and to support the interpretation of FTIR-derived spectral and chemical imaging patterns.

Histological assessment served as the reference for ROI definition and the biological interpretation of FTIR-derived patterns. HE staining provided the general tissue architecture, including vital epidermis, follicular structures, keratinised layers, and inflammatory or reactive tissue changes. PAS and GMS staining highlighted fungal structures within keratinised or follicular compartments. In several cases, fungal elements were found in superficial keratinised tissue or within follicular/infundibular structures, underscoring the need to distinguish fungal infection-associated regions from non-infected keratinised tissue. Representative serial sections stained with GMS, HE, and PAS illustrate the histological basis for identifying fungal infection-associated tissue compartments and for guiding ROI selection for FTIR analysis (Figure 1).

### 3.3. Molecular Species Identification Was Attempted but Remained Inconclusive

Complementary molecular species identification was attempted using DNA extracted from FFPE tissue sections. Despite repeated PCR amplification and subsequent sequencing attempts, sequence data of sufficient quality for reliable species-level identification could not be obtained. Molecular species identification was therefore considered inconclusive and was not used for case classification or ROI definition. All FTIR analyses were instead anchored to histologically identified fungal infection-associated tissue compartments using HE, PAS, and GMS staining.

### 3.4. FTIR Chemical Maps Reveal Spatially Coherent Infection-Associated Patterns

Univariate FTIR chemical maps revealed spatial differences between fungal infection-associated regions, keratinised tissue, and vital epidermis. Across the sample series, the most pronounced contrast was observed within the 900–1300 cm^−1^ fingerprint region, which contains overlapping spectral contributions from carbohydrates, proteins, nucleic acids, and phosphate-containing biomolecules. In contrast, maps dominated by the Amide I and Amide II bands, which primarily reflect protein composition and secondary structure, and by C–H stretching vibrations, which are mainly associated with lipids and keratin-rich structures, provided complementary information on protein-rich and keratinised tissue morphology.

**Figure 2:**
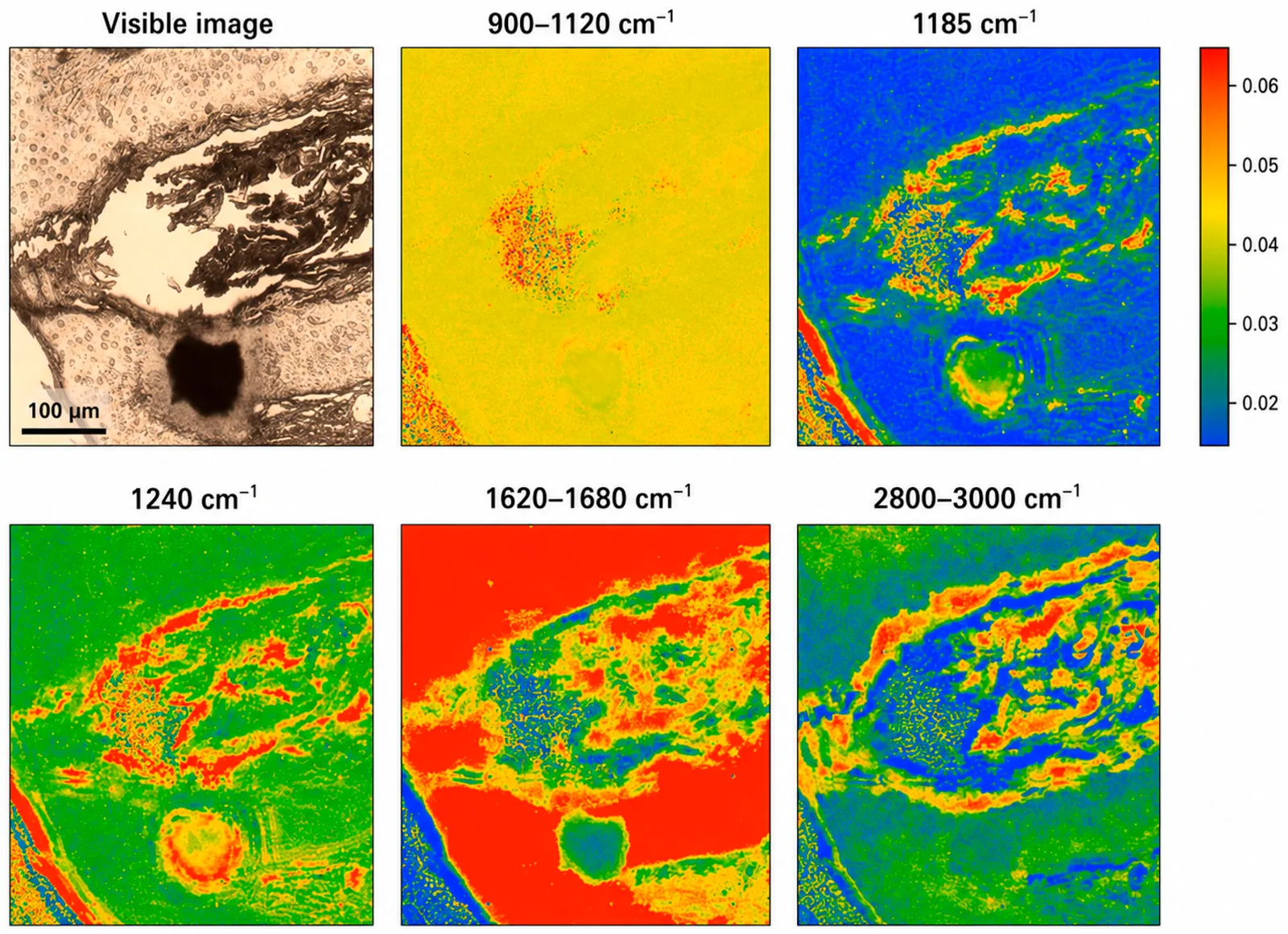
Histology-correlated FTIR chemical imaging of a fungal infection-associated tissue region. Representative GMS-stained reference image and corresponding FTIR chemical maps of a fungal infection-associated skin compartment. The GMS image shows black-stained fungal elements within keratinised tissue, providing the histological reference for the region of interest. The adjacent FTIR chemical maps visualise spatially resolved absorbance patterns from selected spectral regions: 900–1120 cm^−1^, representing the carbohydrate-rich fingerprint region with contributions from C–O and C–C vibrations; 1185 cm^−1^, located within the complex fingerprint region and containing overlapping carbohydrate-, glycoprotein-, and other tissue-associated vibrational contributions; 1240 cm^−1^, reflecting mixed Amide III, C–N, phosphate, and C–O vibrations and contributing to keratin-associated contrast; 1620–1680 cm^−1^, corresponding mainly to the Amide I region related to protein secondary structure and tissue architecture; and 2800–3000 cm^−1^, representing C–H stretching vibrations primarily associated with lipid- and keratin-rich tissue structures. The fingerprint-region maps show pronounced spatial contrast in and around the GMS-positive fungal/keratinised compartment, whereas the Amide I- and C–H-stretching-dominated maps provide complementary information on surrounding protein-rich and keratinised tissue morphology. Colour scales represent relative absorbance intensities within each chemical map and should be interpreted within each panel rather than as directly comparable absolute values across panels. Because the histologically defined fungal infection-associated compartment contains fungal structures together with the surrounding keratinised and epithelial tissue, none of the displayed spectral bands should be interpreted as selectively or exclusively originating from fungal material.

In the following case, maps generated from the 900–1120 cm^−1^, 1185 cm^−1^, and 1240 cm^−1^ regions showed spatially coherent signal patterns corresponding to the GMS-positive follicular compartment. In contrast, the 1620–1680 cm^−1^ Amide I region primarily highlighted protein-rich viable tissue compartments, while the 2800–3000 cm^−1^ CH-stretching region reflected keratin- and lipid-associated tissue morphology. These latter regions were therefore interpreted as complementary tissue-contrast markers rather than fungal-specific maps. A representative histology-correlated FTIR image set from H23_01432 illustrates the spatial correspondence between the GMS-positive fungal/keratinised compartment and selected chemical maps (Figure 2).

**Figure 3:**
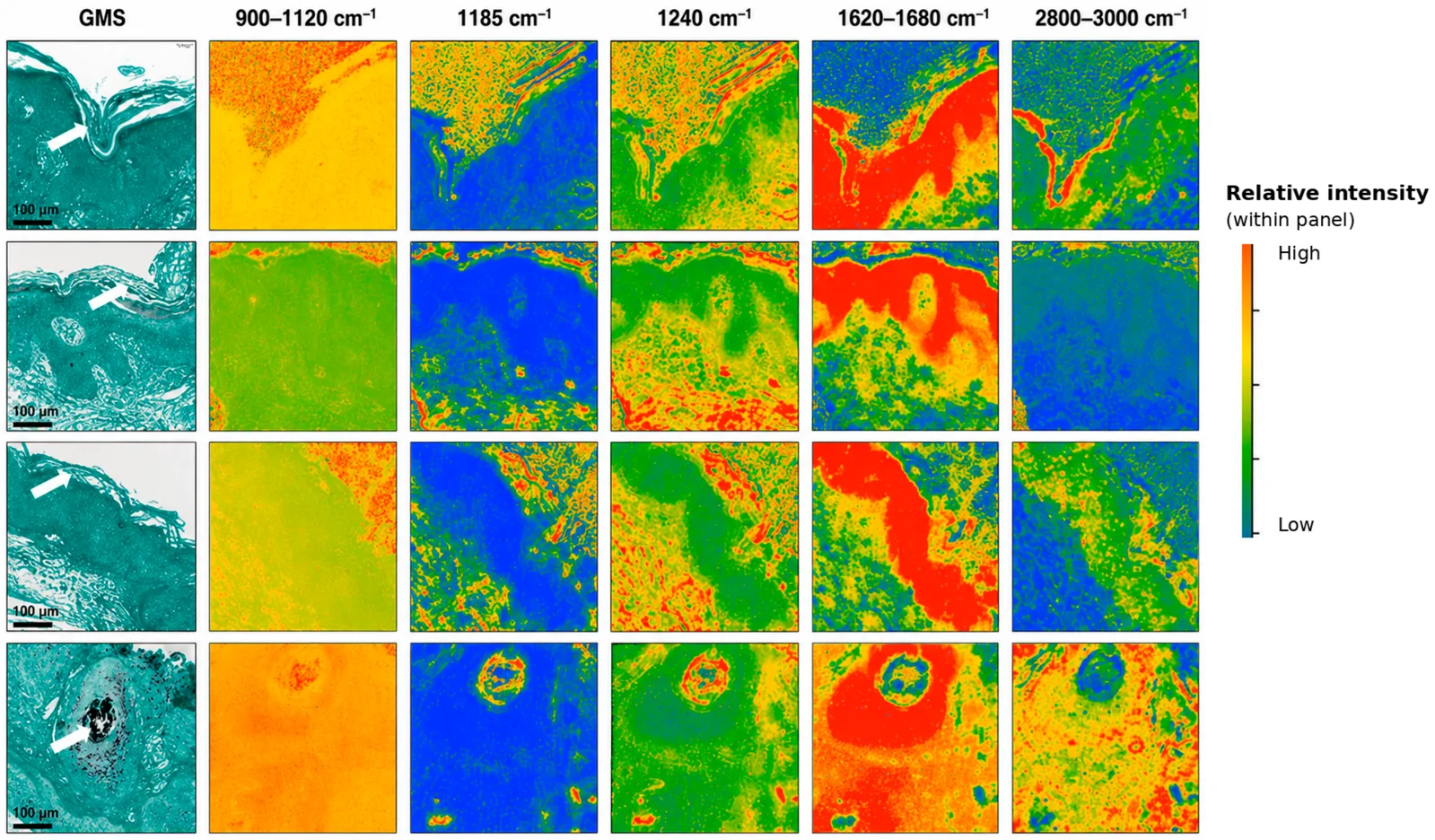
Histology-correlated FTIR chemical mapping of fungal infection-associated skin tissue compartments. Representative GMS-stained histological images and corresponding FTIR chemical maps are shown for multiple fungal infection-associated regions. The first column displays GMS reference images, with white arrows indicating histologically confirmed fungal or keratin-associated regions of interest. The adjacent panels show FTIR chemical maps generated from selected spectral regions: 900–1120 cm^−1^, representing the carbohydrate-rich fingerprint region with contributions from C–O and C–C vibrations; 1120–1200 cm^−1^/1185 cm^−1^, representing a complex fingerprint region with overlapping carbohydrate-, glycoprotein-, and tissue-associated vibrational contributions; 1200–1300 cm^−1^/1240 cm^−1^, reflecting mixed Amide III, C–N, phosphate, and C–O vibrations and contributing to keratin-associated contrast; 1620–1680 cm^−1^, representing the Amide I region related to protein secondary structure and tissue architecture; and 2800–3000 cm^−1^, corresponding to C–H stretching vibrations primarily associated with lipid- and keratin-rich tissue structures. Across the representative regions, the fingerprint-region maps, particularly around 1185 cm^−1^ and 1240 cm^−1^, showed pronounced spatial contrast within keratinised and fungal infection-associated compartments, whereas the Amide I- and C–H-stretching-dominated maps provided complementary information on surrounding protein-rich, lipid-rich, and keratinised tissue morphology. Colour scales represent relative absorbance intensities within each chemical map and should be interpreted within each panel rather than as directly comparable absolute values across different maps or cases. The spatial correspondence with fungal-positive histological compartments indicates an infection-associated spectral pattern but does not establish molecular specificity for fungal material.

To assess whether these spatial patterns were also present across additional fungal infection-associated regions, representative GMS-correlated FTIR chemical maps were evaluated in multiple tissue areas. Across these regions, chemical maps from the 900–1120 cm^−1^ fingerprint region, 1185 cm^−1^, 1240 cm^−1^, 1620–1680 cm^−1^, and 2800–3000 cm^−1^ spectral regions highlighted distinct tissue compartments. The fingerprint-region maps, particularly those around 1185 and 1240 cm^−1^, supported the spatial relevance of carbohydrate-, glycoprotein-, Amide III-, phosphate-, and keratin-associated spectral contributions for fungal infection-associated biochemical contrast, whereas the 1620–1680 cm^−1^ Amide I region and the 2800–3000 cm^−1^ C–H stretching region provided complementary information on protein-rich, lipid-rich, and keratinised tissue morphology (Figure 3).

### 3.5. Spectral Differences Between Fungal Infection-Associated Regions, Keratosis, and Vital Epidermis

**Figure 4:**
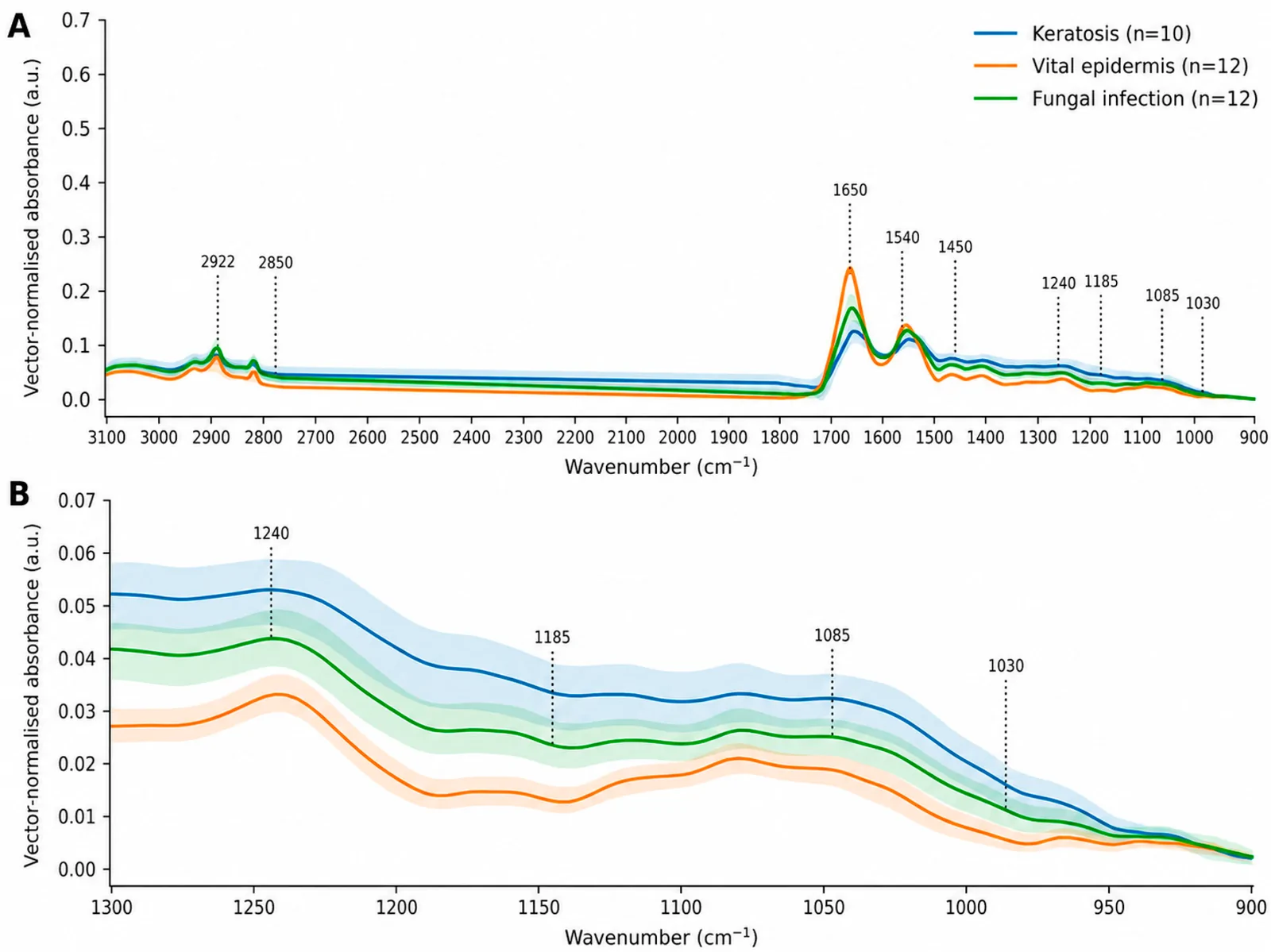
Mean FTIR spectra of fungal infection-associated regions, keratosis, and vital epidermis. (**A**) Overlaid class-wise mean FTIR spectra of keratosis/keratinised tissue, vital epidermis, and fungal infection-associated regions over the analysed spectral range. Where multiple ROIs of the same tissue class were available from one case, ROI-level mean spectra were first averaged to obtain one case-level mean spectrum per class. The resulting case-level sample sizes were *n* = 12 for fungal infection-associated tissue, *n* = 10 for keratosis/keratinised tissue, and *n* = 12 for vital epidermis. Relevant spectral contributions are indicated around 1030, 1085, 1185, 1240, 1450, 1540, 1650, 2850, and 2922 cm^−1^. (**B**) Magnified view of the 900–1300 cm^−1^ fingerprint region, highlighting class-dependent spectral differences around 1030, 1085, 1185, and 1240 cm^−1^. Solid lines represent class-wise means, and shaded areas indicate mean ± SD across case-level mean spectra. The observed differences represent exploratory tissue class-associated spectral patterns and should not be interpreted as fungal-specific diagnostic biomarkers. Quantitative case-level comparisons of the selected spectral bands are reported in Section 3.5.

Mean spectral analysis confirmed class-dependent differences between fungal infection-associated regions, keratosis, and vital epidermis. To facilitate direct visual comparison, the class-wise mean spectra were overlaid across the analysed spectral range, with an additional magnified view of the 900–1300 cm^−1^ fingerprint region (Figure 4). The three tissue classes showed broadly similar overall spectral profiles but differed in relative absorbance intensities and band shapes at several relevant wavenumbers. Prominent descriptive differences were observed within the fingerprint region, particularly around 1030, 1085, 1185, and 1240 cm^−1^. Keratosis showed partially overlapping spectral characteristics with fungal infection-associated regions, consistent with the histological localisation of fungal elements within or adjacent to keratinised tissue. Differences were also observed in the Amide I and Amide II regions around 1650 and 1540 cm^−1^ and in the C–H stretching region around 2850–2920 cm^−1^; however, these regions were interpreted primarily as reflecting differences in protein-, lipid-, and keratin-associated tissue composition rather than fungal-specific signals.

Quantitative case-level analysis confirmed significant tissue-compartment-dependent differences at the principal fingerprint-region bands. In the 10 cases containing all three tissue compartments, overall comparisons were significant at approximately 1030, 1085, 1185, and 1240 cm^−1^ (Friedman tests; FDR-adjusted q = 0.00185, 0.00185, 0.00152, and 0.00152, respectively). In paired comparisons between fungal infection-associated tissue and vital epidermis (*n* = 12), significant differences were observed at approximately 1185 cm^−1^ (q = 0.00110) and 1240 cm^−1^ (q = 0.00110), whereas differences at approximately 1030 and 1085 cm^−1^ did not remain statistically significant after multiple-testing correction (q = 0.151 for both). Fungal infection-associated tissue also differed significantly from keratosis at approximately 1030, 1085, 1185, and 1240 cm^−1^ (q = 0.0293, 0.00586, 0.0132, and 0.0246, respectively), with lower normalized intensities in the fungal infection-associated compartments. Keratosis differed significantly from vital epidermis at all four fingerprint-region bands (all q ≤ 0.00502). These quantitative findings support tissue-compartment-dependent spectral variation within the fingerprint region but also demonstrate the substantial contribution of keratinised tissue to the observed spectral characteristics. Accordingly, these bands should be interpreted as exploratory tissue-compartment-associated spectral features rather than fungal-specific diagnostic biomarkers.

### 3.6. Case-Level Principal Component Analysis of Histologically Defined Tissue Compartments

To account for the dataset’s hierarchical structure and avoid disproportionate weighting from cases contributing many individual spectra, PCA was performed on case-level mean spectra. Individual spectra were first averaged within each ROI, and multiple ROIs belonging to the same tissue class and biological case were subsequently averaged so that each case contributed a maximum of one observation per tissue class.

**Figure 5:**
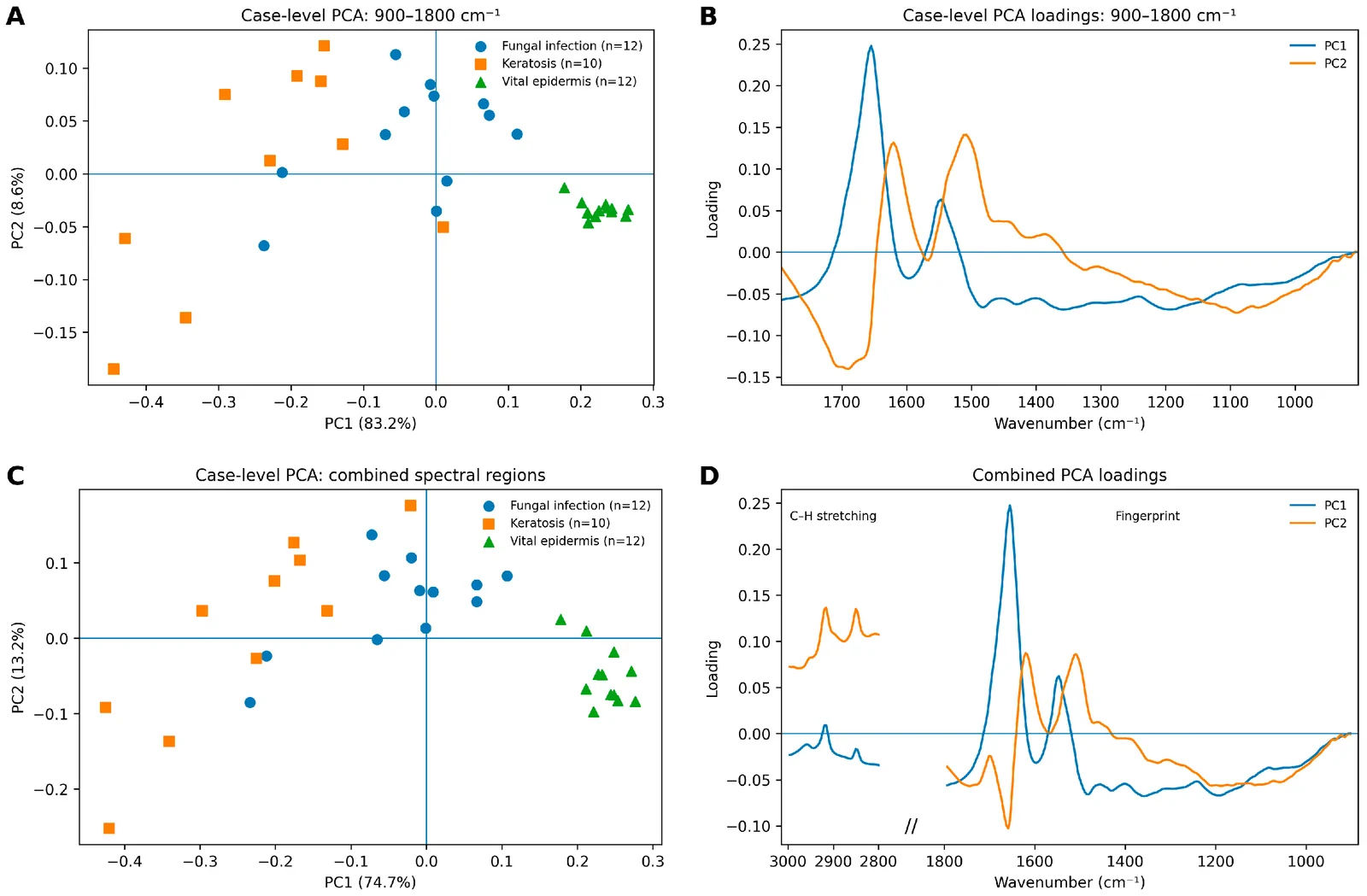
Case-level principal component analysis of histologically defined FTIR tissue compartments. Individual preprocessed spectra were first averaged within each ROI. Where multiple ROIs of the same tissue class were available from the same biological case, ROI-level mean spectra were subsequently averaged so that each case contributed a maximum of one spectrum per tissue class. The case-level dataset comprised fungal infection-associated tissue (*n* = 12 cases), keratosis/keratinised tissue (*n* = 10 cases), and vital epidermis (*n* = 12 cases). (**A**) PCA score plot for the 900–1800 cm^−1^ fingerprint region. PC1 and PC2 explained 83.2% and 8.6% of the total variance, respectively. (**B**) Corresponding PC1 and PC2 loading spectra for the 900–1800 cm^−1^ region. (**C**) PCA score plot for the combined 900–1800 cm^−1^ and 2800–3000 cm^−1^ spectral regions. PC1 and PC2 explained 74.7% and 13.2% of the total variance, respectively. (**D**) Corresponding PC1 and PC2 loading spectra for the combined spectral regions, displayed separately for the fingerprint and C–H stretching segments. Tissue-class labels were used for visualisation only. PCA was exploratory and unsupervised and was not used to derive diagnostic classification performance.

The case-level PCA showed persistent tissue-associated spectral structure after biological aggregation (Figure 5). For the 900–1800 cm^−1^ fingerprint region, PC1 explained 83.2% and PC2 explained 8.6% of the total variance, corresponding to 91.8% cumulative variance. Vital epidermal case-level spectra formed a relatively compact distribution, whereas fungal infection-associated and keratosis spectra showed broader distributions and partial overlap. Thus, the spectral structure observed in the original individual-spectrum analysis persisted when biological cases were given comparable statistical weight.

Examination of the corresponding PC1 and PC2 loading spectra showed that the PCA variance was distributed across multiple spectral regions rather than being determined by a single isolated wavenumber. Prominent contributions were observed particularly in the higher-wavenumber part of the fingerprint region, including the Amide I-associated region, together with additional contributions across the fingerprint spectrum. The loadings were therefore interpreted as indicators of spectral regions contributing to overall tissue-associated variance rather than as fungal-specific molecular markers.

For the combined 900–1800 cm^−1^ and 2800–3000 cm^−1^ dataset, PC1 and PC2 explained 74.7% and 13.2% of the total variance, respectively, for a cumulative 87.9%. The overall distribution was comparable to that of the fingerprint-region PCA, with partial separation of the tissue classes and persistent overlap between fungal infection-associated and keratinised tissue. The isolated C–H stretching-region PCA explained 76.5% and 18.3% of the variance by PC1 and PC2, respectively, and is provided as a supplementary analysis.

ROI-level PCA showed a similar overall structure, with PC1 and PC2 explaining 74.2% and 11.2% of the variance in the combined spectral dataset. The individual-spectrum PCA is retained only to illustrate within-tissue spectral heterogeneity and is not interpreted as representing independent biological observations. Overall, PCA was used as an exploratory visualisation of spectral structure rather than as a diagnostic classification model.

### 3.7. Unsupervised Cluster Analysis of FTIR Images

**Figure 6:**
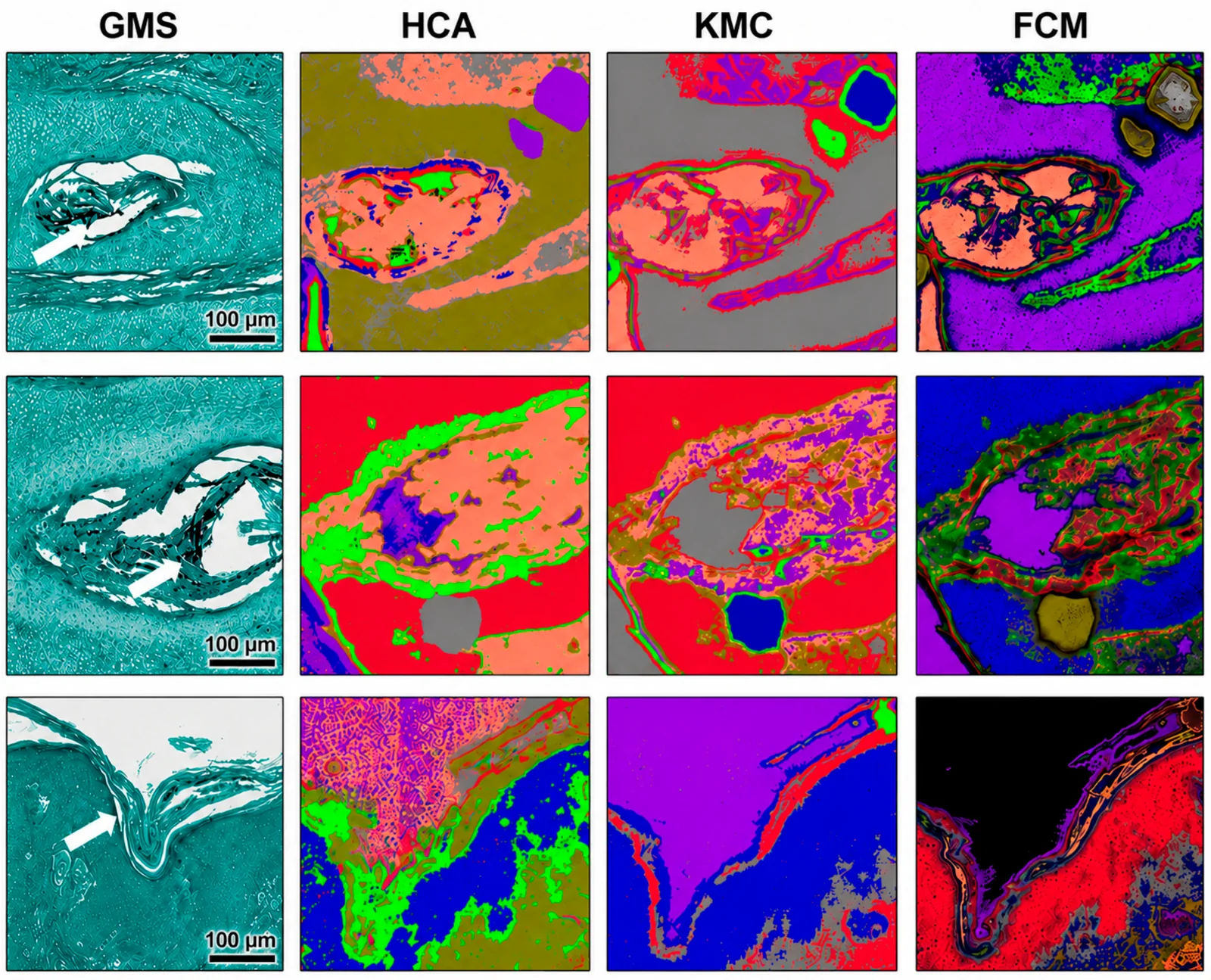
Histology-correlated unsupervised FTIR cluster analysis of fungal infection-associated skin compartments. Representative GMS-stained histological images and corresponding CytoSpec-based unsupervised cluster maps are shown for fungal infection-associated tissue regions. The first column displays GMS reference images, with white arrows indicating histologically confirmed fungal or keratin-associated structures. The adjacent columns show representative cluster maps generated from selected FTIR spectral regions, including the broad fingerprint region 900–1680 cm^−1^, the carbonyl/amide-associated region 1690–1770 cm^−1^, and the lipid/CH-stretching region 2800–3000 cm^−1^. Across the analysed regions, clustering separated spatially coherent tissue compartments corresponding to keratinised material, viable epidermal or follicular structures, stromal tissue, and intralesional fungal/keratin-associated material. Cluster colours represent algorithm-defined spectral classes and do not correspond to fixed biological labels; therefore, all cluster maps were interpreted in relation to the corresponding GMS-stained histological reference images.

Unsupervised clustering was performed using the 900–1680 cm^−1^, 1690–1770 cm^−1^, and 2800–3000 cm^−1^ spectral ranges. Across representative tissue sections, clustering highlighted spatially coherent spectral domains corresponding to morphologically recognisable compartments, including keratinised material, viable epidermal or follicular structures, stromal tissue, and luminal/background areas (Figure 6). Although absolute cluster colours varied across clustering methods and class numbers, the overall spatial organisation of the major tissue compartments was preserved.

In fungal-positive follicular lesions, cluster maps separated the intrafollicular content from the surrounding follicular wall or perifollicular epithelial rim and adjacent stromal tissue. The 900–1680 cm^−1^ fingerprint region provided the most detailed segmentation of fungal/keratin-associated tissue compartments, whereas the 1690–1770 cm^−1^ and 2800–3000 cm^−1^ regions provided complementary information on tissue interfaces and CH-rich keratinised structures. Based on visual comparison with the corresponding histological sections, the seven-class KMC and FCM solutions provided the most interpretable balance between compartment separation and spatial coherence among the evaluated 6-, 7-, and 8-class solutions. Because cluster colours represent algorithm-derived spectral classes, all cluster maps were interpreted only in relation to the corresponding HE, PAS, and GMS-stained sections.

Overall, FTIR chemical imaging identified spatially coherent biochemical differences between fungal infection-associated regions, keratosis, and vital epidermis. Chemical mapping, mean spectral analysis, PCA, and unsupervised clustering consistently indicated that the fingerprint region provided the most informative contrast, while Amide I/II and CH-stretching regions contributed complementary tissue-morphological information.

## 4. Discussion

This proof-of-concept study demonstrates that FTIR chemical imaging can reveal spatially coherent biochemical patterns associated with fungal infection in human skin tissue sections. By combining FTIR spectroscopic imaging with conventional HE, PAS, and GMS staining, we were able to relate spectral differences to histologically defined tissue compartments, including fungal infection-associated regions, keratinised tissue, and vital epidermis. The most informative spectral differences were consistently observed within the 900–1300 cm^−1^ fingerprint region, particularly around 1085, 1185, and 1240 cm^−1^. These findings support the concept that FTIR chemical imaging can complement morphology-based dermatopathology by adding label-free molecular information to spatially resolved tissue assessment.

A critical consideration is the biological meaning of the fungal infection-associated ROI class. These ROIs were defined by histological correlation with PAS- and/or GMS-positive fungal structures but did not represent isolated fungal biomass. Fungal elements in cutaneous infections are frequently embedded within or immediately adjacent to keratinised tissue, and the spatial sampling characteristics of FTIR imaging further result in spectra reflecting the combined biochemical composition of the sampled tissue compartment. Consequently, the measured spectra may include contributions from fungal structures, keratin, viable or altered epithelial components, and the local infection-associated microenvironment. The present dataset therefore does not permit individual spectral features to be assigned uniquely to fungal material. Rather, the differences reported here should be interpreted as biochemical characteristics of histologically confirmed fungal infection-associated tissue compartments.

Histopathology remains central to the diagnosis of clinically atypical fungal infections because it allows for the assessment of fungal elements in their tissue context and provides information on host response, tissue invasion, and associated inflammatory patterns [1,2]. Special stains, such as PAS and GMS, are widely used because fungal structures may be difficult to identify on HE alone, particularly when organisms are sparse, degenerated, or embedded in keratinised material [2,17]. This is highly relevant for cutaneous fungal disease, where fungal elements are frequently located in the stratum corneum, follicular keratin, or other keratin-rich compartments. In the present study, GMS- or PAS-positive structures served as histological anchors for assigning infection-associated regions. Importantly, FTIR-derived maps and multivariate clustering did not replace histology but rather provided complementary biochemical information that could be interpreted in relation to established histological reference stains.

The 900–1300 cm^−1^ fingerprint region contains overlapping contributions from C–O, C–C, C–N, phosphate-related, and other biomolecular vibrations and is sensitive to carbohydrates, nucleic acids, proteins, glycoproteins, and related tissue components. Fungal cell walls contain carbohydrate-rich polymers such as chitin, glucans, and mannans [18], which may contribute to spectra measured in fungal infection-associated compartments. However, keratinised and epithelial tissue contains numerous overlapping absorptions in the same region. The present data therefore do not permit the observed spectral differences to be attributed specifically to fungal cell-wall components. Features around 1085, 1185, and 1240 cm^−1^ are consequently interpreted as exploratory infection-associated tissue characteristics rather than fungal-specific molecular markers. Including keratosis as a separate comparator class was therefore essential for distinguishing infection-associated spectral patterns from general keratinisation.

The quantitative case-level analysis further supports this interpretation. Among the principal fingerprint-region bands, the differences between fungal infection-associated tissue and vital epidermis remained significant after correction for multiple testing at approximately 1185 and 1240 cm^−1^, whereas the corresponding differences at approximately 1030 and 1085 cm^−1^ did not. Importantly, keratosis also showed significant differences from both fungal infection-associated tissue and vital epidermis at these fingerprint-region bands. This finding indicates that the observed spectral contrast is not attributable solely to the presence of fungal material but reflects the biochemical composition of the broader histologically defined tissue compartment, including keratin-associated contributions. The statistical findings therefore reinforce the interpretation of the selected bands as exploratory infection-associated tissue features rather than validated fungal-specific biomarkers.

This distinction is one of the key methodological aspects of the present work. Previous infrared spectroscopy studies have shown that FTIR-based approaches can identify fungal organisms or fungal infections in different contexts, including Candida and dermatophyte species as well as onychomycosis [11,12]. However, many of these studies focused on microbial isolates, nail material, or bulk spectroscopic measurements. In contrast, the present study focused on histologically correlated FTIR chemical imaging of fungal infection-associated structures directly within skin tissue sections. This is important because tissue sections contain heterogeneous biological compartments, including vital epidermis, inflammatory tissue, keratinised layers, follicular structures, and fungal elements. Without histological validation and compartment-specific ROI assignment, spectral differences could be misattributed to fungal organisms when they actually reflect keratinisation or tissue architecture.

The present findings can also be considered in relation to our previous proof-of-concept study applying FTIR microscopy to histologically confirmed scabies in human FFPE skin sections [13]. In that study, FTIR imaging enabled the localisation of histologically identifiable mite-associated structures and showed pronounced spectral contrast of the chitin-rich exoskeleton, particularly around 1072 cm^−1^, compared with surrounding stratum corneum and dermis. Quantitative analysis confirmed significantly higher absorbance at this band in mite-associated regions, and unsupervised clustering supported the spatial delineation of parasite-associated compartments. In contrast, the present fungal application involves a more complex tissue-compartment problem. Fungal elements are frequently embedded within or immediately adjacent to keratinised tissue and may approach the effective spatial resolution of FTIR imaging; consequently, the measured spectra cannot be assigned to isolated fungal material with the same degree of spatial separation. Instead, the present analysis identifies the spectral characteristics of histologically defined fungal infection-associated tissue compartments, with the strongest case-level differences from vital epidermis observed around 1185 and 1240 cm^−1^ and with persistent overlap with keratinised tissue. Thus, while both studies support histology-correlated FTIR imaging as a label-free adjunct for dermatopathological tissue characterisation, the current work extends the approach from the localisation of a relatively well-defined parasite-associated structure to the more heterogeneous biochemical microenvironment of fungal infection.

A further consideration is the spatial resolution of FTIR chemical imaging. Although the projected spatial sampling of the present FPA configuration was approximately 2.7 × 2.7 µm^2^ per pixel, this value does not represent the effective optical resolution, which is wavelength-dependent and diffraction-limited. Individual fungal structures may therefore not always be spatially resolved from the surrounding keratinised or epithelial tissue. Consequently, spectra obtained from fungal infection-associated compartments can represent spatially averaged biochemical contributions from fungal elements and their immediate tissue environment. This further supports the interpretation of the observed spectral differences as infection-associated tissue-compartment characteristics rather than spectra attributable exclusively to fungal organisms.

Our findings also align with the broader development of infrared spectroscopic imaging as a label-free approach for tissue classification and digital histopathology. FTIR spectroscopic imaging has been shown to extract biochemical information from biological samples and generate spatially resolved tissue maps using computational analysis [7]. Earlier work demonstrated the feasibility of infrared spectroscopic imaging for histopathologic recognition, while more recent reviews have emphasised its potential as a complementary method for digital pathology [9,10]. In this context, the present study extends the use of FTIR chemical imaging to fungal infection-associated dermatopathology.

Other optical spectroscopic technologies may provide complementary information to FTIR chemical imaging. Raman spectroscopy probes molecular vibrations through inelastic light scattering and provides molecular contrast complementary to mid-infrared absorption spectroscopy. Using confocal Raman microspectroscopy, Maquelin et al. achieved prediction accuracies of 97–100% for the identification of five clinically relevant Candida species from cultured microcolonies [19]. Surface-enhanced Raman spectroscopy has additionally enabled genus- and species-level differentiation of dermatophyte fungi [20]. Hyperspectral imaging approaches have also been investigated for fungal detection. Using endogenous-fluorescence hyperspectral microscopy, Ye et al. reported sensitivities/specificities of 95%/93% for *Microsporum gypseum* and 94%/93% for *M. canis* [21]. These results demonstrate the potential of complementary spatial-spectral approaches, although differences in specimen type, acquisition principle, and validation strategy preclude a direct performance comparison with the present FTIR study.

Combining univariate chemical maps, PCA-based spectral exploration, and clustering enabled both targeted and unsupervised visualisation of tissue compartments. In particular, the cluster analyses supported the presence of spatially coherent spectral compartments corresponding to histologically defined fungal/keratin-associated structures.

Importantly, the tissue-associated spectral structure observed by PCA persisted after aggregation at the biological case level. In the fingerprint-region analysis, PC1 and PC2 together explained 91.8% of the total spectral variance, while the corresponding components of the combined-region analysis explained 87.9%. Vital epidermal spectra showed the most compact case-level distribution, whereas fungal infection-associated and keratosis spectra remained more heterogeneous and partially overlapping. These findings indicate that the observed PCA structure was not solely a consequence of the large and unequal numbers of individual spectra contributing to each tissue class. Nevertheless, the overlap between fungal infection-associated and keratinised tissue, and the limited number of independent cases, emphasise the exploratory nature of the analysis and preclude interpreting the PCA as evidence of diagnostic discrimination. Accordingly, although diagnostic-performance values from conventional and label-free techniques are provided above as literature benchmarks, sensitivity, specificity, accuracy, and area-under-the-curve metrics were not calculated for the present dataset because no supervised diagnostic classifier was developed or independently validated.

The most robust interpretation of the present data is therefore not that FTIR chemical imaging alone provides a definitive diagnosis of fungal infection, but that it highlights consistent spectral-biochemical patterns within the present dataset associated with histologically confirmed fungal infection-related compartments. Conventional fungal stains remain necessary for biological validation, particularly in proof-of-concept studies with limited case numbers. However, FTIR chemical imaging may add value by providing label-free molecular contrast and by enabling retrospective computational analysis of tissue compartments. Such information could be particularly useful for characterising the biochemical microenvironment of infection, distinguishing infected from non-infected keratinised tissue, and developing future computer-assisted workflows for dermatopathological image analysis.

Several limitations must be considered. First, this study was designed as a proof-of-concept analysis rather than a diagnostic accuracy study, and the number of independent biological cases remains limited.

An additional limitation is the absence of an independent negative-control cohort comprising skin samples without fungal infection. Keratosis/keratinised tissue and vital epidermis served as histologically defined within-case comparator compartments, allowing tissue-associated spectral differences to be evaluated within the same biological specimens. However, these internal comparator regions cannot substitute for independently collected non-infected control tissue. Consequently, the present study cannot determine whether the observed infection-associated spectral characteristics are specific to fungal infection or may also occur in other inflammatory, reactive, or hyperkeratotic skin conditions. Future validation studies should therefore include independent healthy skin controls as well as relevant non-fungal inflammatory and hyperkeratotic disease controls.

Second, individual FTIR spectra were nested within ROIs and biological cases and therefore did not represent independent biological observations. To address this hierarchical structure, PCA was performed at the ROI and case levels, with the case-level analysis used as the primary biological comparison. The persistence of tissue-associated spectral structure after aggregation reduces the likelihood that the observed PCA pattern was driven solely by unequal numbers of spectra per case; however, the present case-level PCA remains exploratory and does not constitute diagnostic validation. Future supervised classification studies should use larger independent cohorts and strictly case-wise validation strategies, such as leave-one-case-out cross-validation. In addition, the standardized post-deparaffinisation drying step at 60 °C may have introduced subtle pre-analytical effects on hydration- or protein-sensitive spectral features. Because the same temperature and exposure time were applied to all sections, this represents a common processing condition across tissue classes; nevertheless, future studies may compare room-temperature drying or controlled desiccation protocols to assess the influence of this step directly. Third, fungal infection-associated ROIs were defined using histological correlation, but exact one-to-one registration between FTIR images and stained serial sections remains technically challenging. In addition, molecular species identification was attempted but could not be successfully completed from the available FFPE-derived DNA aliquots. DNA extraction, quantification, repeated PCR testing, and sequencing attempts were performed; however, the extracted material did not provide sufficient quality and purity for reliable downstream species-level identification. Therefore, molecular species information was not used as a reference standard in the present study. Instead, fungal infection-associated compartments were defined by conventional histological reference staining, particularly PAS and GMS. This limitation is relevant because FTIR-derived spectral patterns in the present study should be interpreted as histology-correlated infection-associated spectral patterns rather than species-specific spectral signatures. Future studies should include prospectively collected material, optimised DNA extraction protocols, and parallel molecular or culture-based species identification to assess whether FTIR chemical imaging can distinguish different fungal taxa.

Furthermore, spectral contributions from fungal organisms and the surrounding tissue cannot be completely separated using the present experimental approach. Fungal elements are frequently embedded within or adjacent to keratinised tissue, and their dimensions may approach or fall below the wavelength-dependent effective spatial resolution of FTIR imaging. Thus, despite the approximately 2.7 × 2.7 µm^2^ projected spatial sampling per FPA pixel, individual spectra may contain mixed contributions from fungal elements, keratinised tissue, adjacent epithelial components, and the local tissue microenvironment. This limitation further supports interpretation of the observed spectral patterns at the level of histologically defined infection-associated tissue compartments rather than as fungal-specific molecular signatures.

Future studies employing higher-resolution correlative imaging, more precise image registration, isolated fungal material, or complementary molecular imaging approaches will be required to determine which spectral components can be attributed specifically to the fungal organisms themselves.

Future work should address these limitations by increasing the number of cases, including defined fungal species when culture or molecular results are available, and validating spectral markers across independent cohorts. It would also be valuable to compare different preprocessing strategies, clustering methods, and classification models using standardised pipelines. Integration with digital histology and more precise image registration may further improve spatial correlation between FTIR-derived biochemical maps and fungal structures identified by GMS or PAS.

In conclusion, this study shows that FTIR chemical imaging can provide spatially resolved biochemical information on fungal infection-associated regions in human skin sections. The strongest spectral contrast was observed in the 900–1300 cm^−1^ fingerprint region, particularly around 1085, 1185, and 1240 cm^−1^. By including keratosis and vital epidermis as separate comparator classes and validating spectral findings against HE, PAS, and GMS staining, the present work provides a histologically grounded framework for interpreting FTIR-derived infection-associated signatures. These findings support FTIR chemical imaging as a promising complementary tool for molecular dermatopathology while emphasising the need for further validation before clinical diagnostic application.

## 5. Conclusions

This proof-of-concept study shows that FTIR chemical imaging can provide label-free, spatially resolved biochemical information on histologically defined fungal infection-associated tissue compartments in human skin. These compartments showed partially distinct spectral characteristics compared with vital epidermis and keratinised tissue, with the most prominent exploratory differences observed in the 900–1300 cm^−1^ fingerprint region, including prominent features around 1085, 1185, and 1240 cm^−1^, with case-level statistical differences between fungal infection-associated tissue and vital epidermis most clearly supported at approximately 1185 and 1240 cm^−1^. Because fungal elements were embedded within or adjacent to keratinised and epithelial tissue, these spectral features cannot be attributed exclusively to fungal material and should be interpreted as exploratory infection-associated tissue signatures. Histological correlation using HE, PAS, and GMS therefore remains essential for biological interpretation. Overall, FTIR chemical imaging may complement conventional dermatopathology by providing additional molecular contrast, but larger studies with independent case-wise validation of supervised classification models are required before diagnostic application can be considered.

## Figures and Tables

**Table 1:** Distribution of independent biological cases, histologically defined ROI datasets, and individual FTIR spectra included in the analysis.

ROI Class	Independent Cases	ROI Datasets	Individual Spectra
Fungal infection-associated regions	12	16	6841
Keratosis/keratinised tissue	10	14	1122
Vital epidermis	12	16	8535
Total unique cases/ROIs/spectra	12	46	16,498

## Data Availability

The data presented in this study are available on request from the corresponding author. The data are not publicly available due to ethical and data-protection restrictions related to human patient-derived tissue and associated patient data.

## Abbreviations

The following abbreviations are used in this manuscript:

CaF_2_	Calcium fluoride
CH	Carbon–hydrogen
C–N	Carbon–nitrogen
C–O	Carbon–oxygen
C–C	Carbon–carbon
FCM	Fuzzy c-means clustering
FFPE	Formalin-fixed, paraffin-embedded
FTIR	Fourier transform infrared
GMS	Grocott methenamine silver
HCA	Hierarchical cluster analysis
HE	Hematoxylin and eosin
KMC	K-means clustering
MCT	Mercury cadmium telluride
PAS	Periodic acid–Schiff
PCA	Principal component analysis
ROI	Region of interest
ROIs	Regions of interest
